# Media-based surveillance of suicide in Türkiye: a mixed-methods analysis of epidemiological patterns, homicide–suicide events, and contextual factors

**DOI:** 10.3389/fpubh.2026.1935064

**Published:** 2026-10-07

**Authors:** Mahmut Talha Uçar, Elif Dönmez, Tulay Ortabag

**Affiliations:** 1 Saray District Health Directorate, Tekirdağ Provincial Health Directorate, Tekirdağ, Türkiye; 2 Department of Oncology Nursing, Hamidiye Faculty of Nursing, University of Health Sciences, Istanbul, Türkiye; 3 Department of Nursing, Faculty of Health Sciences, Istanbul Topkapi University, İstanbul, Türkiye

**Keywords:** document analysis, firearms, homicide–suicide, media reporting, mixed methods, public health, suicide, Turkey

## Abstract

**Background:**

Suicide remains a major public health concern worldwide, yet official mortality statistics often provide limited information regarding the contextual circumstances surrounding suicide events. Media reports provide insights into demographic characteristics, suicide methods, homicide–suicide incidents, and the social contexts associated with these events. This study aimed to examine suicide cases reported in Turkish media using a mixed-methods approach with epidemiological surveillance and qualitative document analysis.

**Methods:**

A convergent mixed-methods design was employed. Suicide-related news reports published in Turkish media between January and December 2025 were prospectively monitored and screened. After removing duplicate reports, 688 suicide cases were included in the quantitative analysis. Descriptive and inferential statistical analyses were conducted to examine demographic characteristics, suicide methods, geographic distribution, and homicide–suicide patterns. Qualitative document analysis was performed on contextual narratives from 179 suicide cases. Quantitative and qualitative findings were integrated during interpretation.

**Results:**

Among the 688 media-reported suicide cases, 76.7% were male, 15.7% were female, and sex was not reported for the remaining 7.6%. Firearms (43.2%) and hanging (30.4%) were the most frequently reported suicide methods. Homicide–suicide was identified in 16.1% of cases and was significantly more common among males than females (20.3% vs. 2.8%, unadjusted OR = 8.90, 95% CI: 2.77–28.58, *p* < 0.001). Suicide method was significantly associated with homicide–suicide status (*p* < 0.001). Among suicides involving firearms, 30.6% were classified as homicide–suicide. Within the media-reported sample, the proportion of cases classified as homicide–suicide differed significantly across regions. Qualitative analysis identified one descriptive event/legal category—violence, homicide–suicide event characteristics, and legal circumstances (31.8%)—and four contextual themes: physical and psychological health problems and addictions (26.3%); family and intimate relationship conflicts (24.6%); economic and occupational difficulties (15.6%); and social exclusion and social vulnerability (1.7%). Integration of findings indicated that homicide–suicide events were frequently embedded within narratives of domestic violence, relationship conflict, and legal problems, whereas psychological distress and economic hardship emerged as common contextual factors across suicide cases.

**Conclusion:**

Media-reported suicide cases in Türkiye demonstrated distinct epidemiological and contextual patterns that should be interpreted within the context of media reporting rather than as representative of all suicide deaths in the country. Combining epidemiological surveillance with qualitative analysis provides complementary contextual information that may inform suicide prevention policies, responsible media reporting practices, and future research integrating official mortality data.

## 1 Introduction

Suicide remains one of the most significant public health challenges worldwide, accounting for substantial mortality, disability, and social burden across populations. According to the World Health Organization (WHO), approximately 727,000 people died by suicide in 2021, representing more than one in every 100 deaths globally. Suicide is also among the leading causes of death among young people aged 15–29 years, highlighting its profound impact on individuals, families, communities, and healthcare systems (1, 2). Despite advances in mental health care and suicide prevention initiatives, suicide continues to pose a major global health concern, reflecting the complex interaction of individual, social, economic, and environmental factors.

Suicide is increasingly conceptualized as a public health outcome influenced not only by psychiatric disorders but also by broader social determinants of health, including unemployment, income insecurity, housing conditions, childhood adversity, social exclusion, and access to healthcare services (3, 4). Although mental disorders remain among the strongest risk factors for suicidal behavior, growing evidence suggests that broader structural factors—including socioeconomic disadvantage, unemployment, debt burden, social isolation, relationship breakdown, exposure to violence, and limited access to support services—also play critical roles in suicide risk (3, 5). Recent studies have highlighted the role of socioeconomic disadvantages, occupational vulnerability, and financial hardship in shaping suicide risk. Men experiencing socioeconomic adversity appear to be at particularly elevated risk, while debt burden has been consistently associated with suicidality and poor mental health outcomes (6, 7). Consequently, understanding suicide requires approaches that extend beyond individual-level explanations and incorporate the broader social and contextual environments in which suicidal behaviors occur.

Although global age-standardized suicide rates have declined in many regions over recent decades, important demographic and geographic disparities persist. Men consistently die by suicide at higher rates than women, and considerable variations exist across countries with respect to suicide mortality, age distributions, and associated risk factors (8). Access to lethal means remains an important determinant of suicide mortality, with firearms, hanging, and poisoning among the most commonly reported methods worldwide (2). These variations underscore the importance of country-specific surveillance systems and context-sensitive suicide prevention strategies that are informed by local epidemiological patterns and social contexts (2).

In Türkiye, suicide has emerged as an increasingly important public health issue. According to the Turkish Statistical Institute (TURKSTAT), 4,460 individuals died by suicide in 2024, corresponding to a crude suicide rate of 5.22 per 100,000 population. Like global trends, males account for the majority of suicide deaths in Türkiye. Previous national studies have identified significant associations between suicide mortality and factors such as gender inequality, socioeconomic conditions, family structure, and regional disparities. Furthermore, recent research has emphasized the growing importance of suicide prevention among children and adolescents, drawing attention to the need for evidence-based interventions tailored to different population groups (9–11). Recent analysis of official Turkish Statistical Institute (TURKSTAT) mortality data (2007–2022) demonstrated sex-specific trends in suicide mortality, with increasing rates among males and a recent increase among females after 2019. The same study also reported rising suicide mortality among young adults aged 15–29 years (12).

Reliable surveillance systems constitute a cornerstone of effective suicide prevention. Official mortality statistics provide essential information regarding suicide rates, demographic characteristics, and temporal trends (2). However, these databases often provide limited information regarding the social and contextual circumstances surrounding suicide events. Factors such as interpersonal conflicts, intimate partner violence, economic difficulties, legal problems, family disputes, and other precipitating life stressors are often not systematically documented in routine mortality statistics, highlighting the value of complementary data sources such as media reports (13, 14).

In recent years, media reports have increasingly been recognized as a valuable supplementary source of information for suicide surveillance. News reports frequently contain detailed descriptions of the circumstances surrounding suicide events, including information regarding family relationships, economic stressors, legal issues, mental health concerns, and social contexts that are often unavailable in official datasets. Consequently, media-based surveillance may contribute to a more comprehensive understanding of suicide by capturing contextual information that complements traditional epidemiological data. Media reports are increasingly used as secondary data sources to examine suicide patterns and contextual characteristics, particularly when timely surveillance data or detailed case-level information are unavailable. Although official mortality statistics remain the primary source for monitoring suicide epidemiology, media reports can provide complementary information on the circumstances surrounding suicide events. However, media coverage reflects journalistic selection and reporting practices rather than the complete epidemiology of suicide. Therefore, findings derived from media-based surveillance should be interpreted as reflecting reported cases and contextual narratives rather than the full distribution of suicide characteristics in the population (1, 15).

Beyond their role as information sources, media reports may themselves influence suicidal behavior. A substantial body of evidence has demonstrated associations between suicide reporting and subsequent suicide rates. Detailed descriptions of suicide methods, sensationalized headlines, repetitive coverage, and prominent reporting have been associated with increases in suicidal behavior, a phenomenon commonly referred to as the “Werther effect” (13). Conversely, responsible reporting practices that emphasize coping strategies, recovery, resilience, and help-seeking behaviors may contribute to protective effects, known as the “Papageno effect” (16). In response to these findings, the World Health Organization and other international organizations have developed evidence-based guidelines for responsible suicide reporting and have emphasized the importance of monitoring media coverage of suicide-related events (14).

The role of media reporting is particularly relevant in Türkiye. Recent evaluations of Turkish media have indicated that suicide-related news reports frequently contain explicit descriptions of suicide methods, sensationalized language, and limited information regarding prevention resources or mental health support services. Such reporting practices may not fully align with international recommendations and may contribute to potentially harmful reporting effects. Moreover, analyses of high-profile suicide incidents and suicide clusters in Türkiye have raised concerns regarding possible contagion and copycat effects associated with intensive media coverage (15, 17). These findings underscore the need for continued examination of suicide reporting practices within the Turkish media landscape.

An especially severe but understudied form of lethal violence is homicide–suicide, defined as the killing of one or more individuals followed by the perpetrator’s suicide. Homicide–suicide events frequently occur in the context of intimate partner violence, family conflicts, firearm access, and severe psychosocial stressors (18). These incidents represent a unique intersection between suicide prevention, violence prevention, and public safety and often generate considerable public and media attention. Despite their public health significance, homicide–suicide events remain insufficiently investigated in many countries, including Türkiye, and are not routinely examined within official surveillance systems (18).

Although previous research in Türkiye has primarily focused on official mortality statistics and quantitative analyses of suicide trends, relatively little is known about the epidemiological characteristics and contextual narratives of suicide cases reported in the media. Furthermore, few studies have integrated quantitative epidemiological analyses with qualitative exploration of contextual factors associated with suicide events. A mixed-methods approach may therefore provide a more comprehensive understanding of suicide by combining epidemiological surveillance with qualitative examination of the social, economic, psychological, and relational circumstances described in media reports. Specifically, the combination of prospective daily media monitoring, quantitative characterization of homicide–suicide events, and qualitative analysis of case-level contextual narratives remains underexamined in Türkiye.

Therefore, this study aimed to examine suicide cases reported in Turkish media using a convergent mixed-methods design. Specifically, the study sought to (1) describe the epidemiological characteristics of suicide cases, including demographic features, suicide methods, geographic distribution, and homicide–suicide patterns, and (2) identify and analyze contextual factors associated with suicide through qualitative document analysis of media reports. By integrating quantitative and qualitative findings, this study aims to contribute to suicide prevention research, public health surveillance, and the development of evidence-informed recommendations for responsible media reporting in Türkiye.

## 2 Methods

### 2.1 Study design

The present study used a convergent mixed-methods methodology to analyze suicide instances reported in Turkish online news media in 2025. Quantitative studies were used to describe the epidemiological characteristics of suicide cases, demographic factors, regional distribution, suicide methods, and homicide-suicide events. Qualitative theme analysis was also conducted to analyze the contextual elements and stories of suicide as reported in the media. The results of the two components were merged in the interpretation process to reach a thorough understanding of suicide instances in the Turkish news coverage.

### 2.2 Data collection

Data were collected through daily monitoring of Google Alerts using the Turkish keywords “İntihar” (“Suicide”) and “Kendini Öldürdü” (“Killed Himself/Herself”). Google Alerts notifications were checked daily from January 1, 2025, to January 15, 2026. Data collection continued until January 15, 2026. Only suicide episodes that occurred between January 1 and December 31, 2025, were included in the study. The prolonged collection period ensured the capture of news reports published after the occurrence of suicide events during 2025.

Google Alerts aggregates news reports from a wide range of national, regional, and local media sources. Multiple media reports frequently described the same suicide event. Rather than treating these reports as separate observations, all reports referring to the same event were reviewed together to obtain the most complete information available. Information extracted from these reports was consolidated into a single case record representing one unique suicide event, and eligibility assessment was subsequently performed at the case level. Consequently, a large number of media reports were reviewed during the surveillance period, substantially exceeding the number of distinct suicide cases included in the study. Thus, the unit of analysis throughout the quantitative analyses was the unique suicide case rather than the individual media report. The overall process of media report identification, screening, consolidation of reports referring to the same suicide event, and final case selection is summarized in Figure 1. Briefly, 27,800 media reports were identified; 24,900 duplicate or syndicated reports referring to previously identified events were consolidated or removed before case-level screening. Of 2,900 candidate case records, 2,212 were excluded: 480 non-fatal suicide attempts, 145 homicides without subsequent suicide, 620 events outside Türkiye, 185 historical or recirculated reports, 170 deaths of ambiguous cause, 105 fictional or music-related reports, 195 suicide-prevention or policy reports, and 312 reports with insufficient information or irrelevant content. This process yielded 688 eligible unique suicide cases, of which 179 contained contextual information.

**Figure 1:**
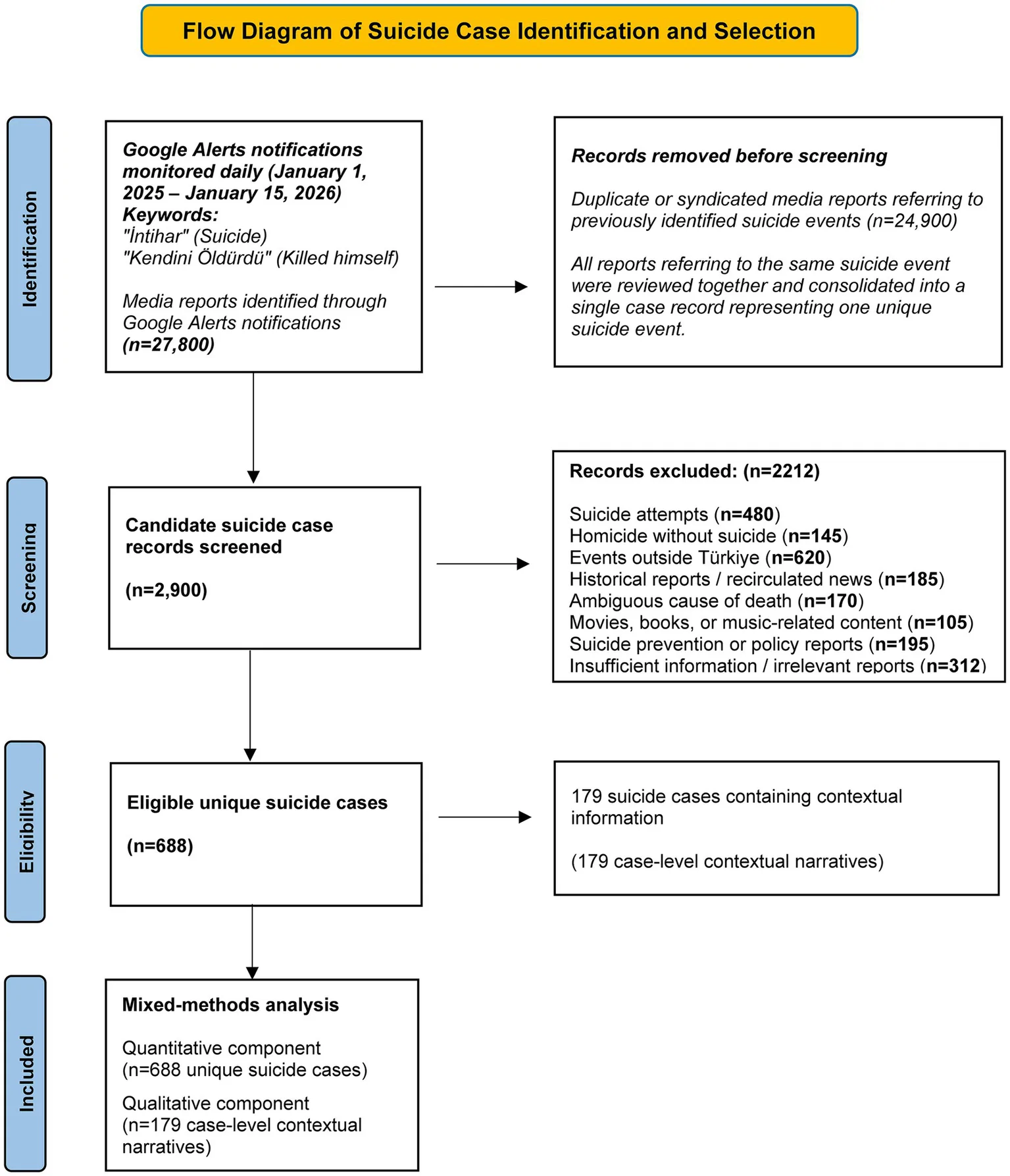
Flow diagram of suicide case identification and selection.

The classification of suicide techniques was determined according to the classification used by the Turkish Statistical Institute (TURKSTAT, TÜİK) which allows comparison with national mortality statistics.

### 2.3 Inclusion criteria

(1) News reports were included if they satisfied all of the following criteria:
(2) The event took place on the territory of Türkiye.
(3) The event took place between January 1 and December 31, 2025.
(4) The report detailed a suicide death.
(5) The report contained sufficient information to identify the occurrence and derive at least the basic epidemiological characteristics.
(6) The report was a unique suicide occurrence following duplication examination.

When several reports described the same incident, all reports were evaluated and combined into a single case record.

### 2.4 Exclusion criteria

Reports were excluded if they met one or more of the following:

(1) The incident took place outside Türkiye.
(2) The report included an incident of self-harm or suicide attempt that did not result in death.
(3) The story was about homicide not followed by suicide.
(4) The report documented suicide attacks, suicide bombings or other acts of political violence by terrorists.
(5) The report relied on historical occurrences, past case reports, anniversaries, retrospective news pieces or cases that were not part of the period of research.
(6) Instead, the report focused more on suicide prevention initiatives, awareness actions, policy discussions, instructional content, or statistics summaries rather than a specific suicide event.
(7) The report mentioned novels, films, television programs, songs, metaphoric uses of the term suicide or other content not tied to an occurrence.
(8) Circumstances of death were reported as ambiguous, suspicious or under investigation and information available was insufficient to determine whether the incident was a suicide death.
(9) There is not enough information in the report to determine the event or if it qualified for inclusion.
(10) The report was a duplicate of an earlier one.

If several reports referred to the same occurrence, only one consolidated case record was maintained in the final data set.

### 2.5 Data extraction variables

A standardized data extraction form was developed prior to data collection. For each eligible suicide case, information was extracted from the available news reports and recorded in a structured database.

The extracted variables included demographic characteristics (sex and age), geographic information (province and statistical region), date of the event, suicide method, location of the incident, presence and type of suicide note, and whether the event involved homicide–suicide. When homicide–suicide was identified, the number of victims and the relationship between the victim(s) and the perpetrator were additionally recorded.

Information regarding contextual circumstances described in the news reports was also extracted. These narratives included reported references to interpersonal conflicts, family or relationship problems, economic difficulties, occupational stressors, physical illnesses, mental health problems, legal issues, criminal investigations, social exclusion, and other circumstances mentioned in connection with the suicide event.

Because multiple media outlets frequently reported the same incident, all identified records underwent a duplicate review process. Cases referring to the same event were identified using available personal identifiers (e.g., name, initials, age, sex), temporal information (date of the event), geographic information (province, district, or specific location), suicide method, and contextual details. Reports determined to describe the same incident were merged into a single case record.

For cases in which personal identifiers were unavailable, event uniqueness was assessed using a combination of location, date, suicide method, demographic characteristics, and narrative descriptions. When uncertainty existed regarding whether multiple reports referred to the same event, all available reports were reviewed collectively and a consensus decision was made by the research team before inclusion.

The final dataset consisted of unique suicide cases rather than individual news reports.

### 2.6 Variable definitions

The primary unit of analysis was the individual suicide case. Cases were classified according to sex, age group, geographic region, suicide method, suicide note status, location of the incident, and homicide–suicide status.

Sex was categorized as male, female, or not reported. Age was categorized as ≤18 years, 19–64 years, and ≥65 years. The ≤18-year category was selected in accordance with the legal definition of childhood under Turkish legislation, whereas the ≥65-year category was defined according to the Turkish Statistical Institute (TURKSTAT) classification for older adults. These age categories were also considered appropriate for public health reporting and epidemiological comparisons.

Suicide methods were classified according to the Turkish Statistical Institute (TURKSTAT) categorization and included firearms, hanging, jumping from height, drowning, chemical ingestion, sharp instruments, burning, gas exposure, train/vehicle-related methods, and other methods. For selected analyses, methods were additionally grouped into five broader categories: firearms, hanging, jumping from height, other methods, and not reported. This grouping was performed because several of the detailed TURKSTAT categories contained relatively few observations. Combining these low-frequency methods into a single category reduced sparse cell counts, improved the stability of the contingency tables, and facilitated more reliable statistical comparisons while preserving the most frequently reported suicide methods as separate categories. Homicide–suicide was defined as an incident in which an individual intentionally killed one or more persons and subsequently died by suicide as part of the same event or sequence of events. Cases involving only suicide without harm to others were classified as non–homicide–suicide. Cases were classified as homicide–suicide only when media reports clearly indicated that the homicide and the subsequent suicide were directly linked and occurred as part of the same incident or an uninterrupted sequence of events. Cases in which the temporal relationship or linkage between the homicide and suicide could not be determined with sufficient certainty from the available reports were not classified as homicide–suicide. The study recorded the method of suicide but did not systematically record the weapon or method used in the preceding homicide. Therefore, the observed association between firearm use and homicide–suicide refers to the suicide method among homicide–suicide cases rather than the weapon used in the homicide itself. In homicide–suicide cases, victims were categorized according to their relationship with the perpetrator, including spouse, child, intimate partner, former partner, family member, friend, colleague, neighbor, and other relationships.

Geographic location was classified according to the NUTS-1 statistical regional classification used in Türkiye. Regions included Istanbul, Western Marmara, Aegean, Eastern Marmara, Western Anatolia, Mediterranean, Central Anatolia, Western Black Sea, Eastern Black Sea, Northeastern Anatolia, Middle East Anatolia, and Southeastern Anatolia.

Suicide note status was categorized as present or not reported/unknown. When present, notes were further classified as written notes or social media posts based on information reported in the news source.

Contextual factors reported in media narratives were extracted and analyzed qualitatively. These factors referred to circumstances, events, or conditions described in news reports as being associated with the suicide event, including interpersonal conflicts, economic difficulties, physical or mental health problems, legal issues, and social stressors. These variables were not interpreted as verified causal factors but rather as reported contextual narratives presented in the media.

### 2.7 Statistical analysis

Quantitative data were analyzed using IBM SPSS Statistics for Windows, Version 27.0 (IBM Corp., Armonk, NY, USA).

Categorical variables were summarized using frequencies and percentages, whereas continuous variables were presented as mean ± standard deviation (SD) or median and interquartile range (IQR), as appropriate. The distribution of continuous variables was evaluated using graphical methods and tests of normality.

Associations between categorical variables were primarily assessed using Pearson’s chi-square test. When the assumptions of the chi-square test were not fully satisfied because of sparse expected cell frequencies, the Fisher–Freeman–Halton exact test was used. For the principal association between sex and homicide–suicide status, an unadjusted odds ratio (OR) with a 95% confidence interval (CI) was calculated by comparing male with female cases; cases for which sex was not reported were excluded from this contrast. Regional analyses were comparisons within the media-reported sample and were not interpreted as estimates of regional incidence or population-level risk.

Since age was not normally distributed, comparisons involving more than two groups were performed using the Kruskal–Wallis test, whereas comparisons between two independent groups (homicide–suicide vs. suicide-only) were performed using the Mann–Whitney *U* test.

All statistical tests were two-sided, and a *p*-value of <0.05 was considered statistically significant.

For the qualitative component, thematic analysis was conducted according to the approach described by Braun and Clarke. Contextual narratives extracted from news reports were coded inductively, grouped into categories, and organized into overarching themes. Quantitative and qualitative findings were subsequently integrated during interpretation within a convergent mixed-methods framework.

### 2.8 Qualitative document analysis

A qualitative document analysis was conducted to explore the contextual factors associated with suicide events reported in Turkish media. Document analysis is a systematic procedure for reviewing, examining, and interpreting textual data to elicit meaning and develop empirical knowledge (19). News reports frequently contain narrative descriptions of potential circumstances surrounding suicide events, including interpersonal conflicts, economic difficulties, health problems, legal issues, and social stressors. These narrative statements were extracted verbatim and treated as qualitative data units for analysis. The qualitative analysis was guided by Braun and Clarke’s thematic analysis (20, 21).

Among the 688 unique suicide cases included in the study, contextual information regarding the circumstances or possible reasons surrounding the suicide event was identified in 179 cases. These contextual narratives reflected circumstances reported by the media and were not interpreted as verified causes of suicide. The contextual narratives extracted from these reports constituted the qualitative dataset. After all available reports referring to the same suicide case had been reviewed together, the contextual information for each case was synthesized into a single case-level narrative. Two researchers (MTU and ED) independently reviewed these narratives and generated initial inductive codes through manual coding. Following independent coding, the researchers compared and discussed their coding decisions, and conceptually similar codes were grouped into broader categories and subsequently organized into overarching themes through consensus. The thematic structure was developed iteratively by the research team. Although a single case-level narrative could contain multiple contextual issues and therefore receive multiple initial descriptive codes, each of the 179 case-level narratives was ultimately assigned to one mutually exclusive primary analytic category based on its dominant contextual meaning. Accordingly, the analytic-category frequencies presented in Table 1 represent the number of suicide cases containing contextual information rather than the total number of coded statements.

**Table 1:** Descriptive event/legal category and contextual themes identified from 179 case-level contextual narratives.

Analytic category	*n*	%	Representative quotation
Descriptive event/legal category: Violence, Homicide–Suicide Event Characteristics, and Legal Circumstances	57	31.8	“After killing his wife, he ended his own life using the same firearm.”
Contextual theme: Physical and Psychological Health Problems and Addictions	47	26.3	“He was reported to have been experiencing psychological problems.”
Contextual theme: Family and Intimate Relationship Conflicts	44	24.6	“He was reported to have become emotionally distressed after his girlfriend wanted to end the relationship.”
Contextual theme: Economic and Occupational Difficulties	28	15.6	“He was reported to have experienced severe emotional distress due to an inability to repay his debts.”
Contextual theme: Social Exclusion and Social Vulnerability	3	1.7	“He was reported to have felt socially excluded by those around him.”

To enhance the trustworthiness of the analysis, coding and theme development were performed independently by two researchers (MTU and ED). Discrepancies in coding and interpretation were resolved through discussion until consensus was reached. The final thematic framework was subsequently reviewed and agreed upon by the research team. Credibility and dependability were strengthened through researcher triangulation, iterative review, and transparent documentation of coding decisions, consistent with the criteria proposed by Lincoln and Guba (22). Through this iterative process, one descriptive event/legal category and four contextual themes were identified: (1) Violence, Homicide–Suicide Event Characteristics, and Legal Circumstances; (2) Physical and Psychological Health Problems and Addictions; (3) Family and Intimate Relationship Conflicts; (4) Economic and Occupational Difficulties; and (5) Social Exclusion and Social Vulnerability.

### 2.9 Ethical considerations

This study was based exclusively on publicly available news reports obtained from online media sources. No direct contact was established with individuals, and no identifiable personal information was retained in the analytical dataset. All data were analyzed in aggregate form. Given the use of publicly available secondary data, formal ethical approval was not required under national regulations.

## 3 Results

A total of 688 suicide cases reported in Turkish media were included. Males accounted for 76.7% (*n* = 528) of cases, females for 15.7% (*n* = 108), while sex was not reported in 7.6% (*n* = 52). Among cases with available age data (*n* = 507), most individuals were aged 19–64 years (87.0%, *n* = 441). Firearms were the most frequently reported suicide method (43.2%, *n* = 297), followed by hanging (30.4%, *n* = 209). Other methods included jumping from height (6.7%, *n* = 46), train- or vehicle-related incidents (2.6%, *n* = 18), sharp instruments (1.7%, *n* = 12), drowning (1.6%, *n* = 11), and chemical ingestion (1.5%, *n* = 10). Homicide–suicide was identified in 16.1% (*n* = 111) of cases (Table 2).

**Table 2:** Factors associated with homicide–suicide (*N* = 688).

Variable	Category	Non-homicide-suicide *n* (%)	Homicide-suicide *n* (%)	Total (*n*)	*p*-value
Sex	Male	421 (79.7)	107 (20.3)	528	<0.001^†^
	Female	105 (97.2)	3 (2.8)	108	
	Not reported	51 (98.1)	1 (1.9)	52	
Age groups^*^	≤18 years	35 (97.2)	1 (2.8)	36	0.040^‡^
	19–64 years	367 (83.2)	74 (16.8)	441	
	≥65 years	24 (80.0)	6 (20.0)	30	
Statistical region (NUTS-1)	Istanbul	59 (79.7)	15 (20.3)	74	<0.001^‡^
	Western Marmara	20 (80.0)	5 (20.0)	25	
	Aegean	111 (84.1)	21 (15.9)	132	
	Eastern Marmara	91 (92.9)	7 (7.1)	98	
	Western Anatolia	16 (59.3)	11 (40.7)	27	
	Mediterranean	46 (67.6)	22 (32.4)	68	
	Central Anatolia	34 (75.6)	11 (24.4)	45	
	Western Black Sea	55 (90.2)	6 (9.8)	61	
	Eastern Black Sea	23 (100.0)	0 (0.0)	23	
	Northeastern Anatolia	8 (88.9)	1 (11.1)	9	
	Middle East Anatolia	38 (97.4)	1 (2.6)	39	
	Southeastern Anatolia	76 (87.4)	11 (12.6)	87	
Method of suicide	Hanging	202 (96.7)	7 (3.3)	209	<0.001^‡^
	Chemicals	10 (100.0)	0 (0.0)	10	
	Jumping	45 (97.8)	1 (2.2)	46	
	Drowning	11 (100.0)	0 (0.0)	11	
	Firearms	206 (69.4)	91 (30.6)	297	
	Burning	3 (100.0)	0 (0.0)	3	
	Sharp instrument	7 (58.3)	5 (41.7)	12	
	Gas	2 (100.0)	0 (0.0)	2	
	Train/vehicle	18 (100.0)	0 (0.0)	18	
	Not reported	73 (91.3)	7 (8.8)	80	

^*^Age information was available for 507 (73.7%) of the 688 suicide cases. Accordingly, all analyses involving age were performed using the available cases only, and the corresponding denominators are reported where applicable.

^†^*p* values were calculated using Pearson’s chi-square test.

^‡^*p* values were calculated using the Fisher–Freeman–Halton exact test because Pearson chi-square assumptions were not fully satisfied owing to sparse expected cell counts.

Cases were most frequently reported from the Aegean region (19.2%, *n* = 132), Eastern Marmara (14.2%, *n* = 98), and Southeastern Anatolia (12.6%, *n* = 87). The geographic distribution of suicide cases across the provinces of Türkiye is illustrated in Figure 2. The highest number of cases was observed in June (15.8%, *n* = 109), followed by August (13.8%, *n* = 95) and May (10.9%, *n* = 75). The monthly distribution of suicide cases is shown in Figure 3. Home was the most common location of suicide (49.0%, *n* = 337). A suicide note was reported in 6.4% (*n* = 44) of cases, including written notes (4.1%, *n* = 28) and social media posts (2.3%, *n* = 16). Individuals involved in homicide–suicide incidents were significantly older than those who died by suicide alone (mean age 42.7 ± 14.2 vs. 35.3 ± 14.8 years; median [IQR]: 38.0 [33.5–54.0] vs. 31.0 [24.0–44.0] years, Mann–Whitney U test, *p* < 0.001). Homicide–suicide was significantly more common among males than females (20.3% vs. 2.8%; unadjusted OR = 8.90, 95% CI: 2.77–28.58, *p* < 0.001). Within the media-reported sample, the proportion classified as homicide–suicide differed across regions, with the highest sample-specific proportions observed in Western Anatolia (40.7%) and the Mediterranean region (32.4%; *p* < 0.001). These percentages represent comparisons within the assembled media sample and should not be interpreted as regional incidence or risk estimates. Suicide method was significantly associated with homicide–suicide status (Fisher–Freeman–Halton exact test, *p* < 0.001). Among firearm-related suicides, 30.6% involved homicide–suicide (Table 2). Suicide methods differed significantly by sex (*p* < 0.001). Firearms were the predominant suicide method among males (49.6%), whereas hanging (39.8%) and jumping from height (18.5%) were relatively more common among females (Table 3). The secondary grouped-method analysis yielded a significant age difference (*p* = 0.017); however, the original detailed method categories did not (*p* = 0.087), and the grouped result should be interpreted cautiously because low-frequency categories were combined to address sparse cell counts (Table 4). Among the 134 victims identified in homicide–suicide incidents, the mean age was 32.8 ± 16.7 years. The value 134 represents the number of victims, not incidents; some of the 111 homicide–suicide incidents involved multiple victims. Spouses (25.4%) were the most common victims, followed by children (13.4%) and girlfriends (8.2%; Table 5).

**Figure 2:**
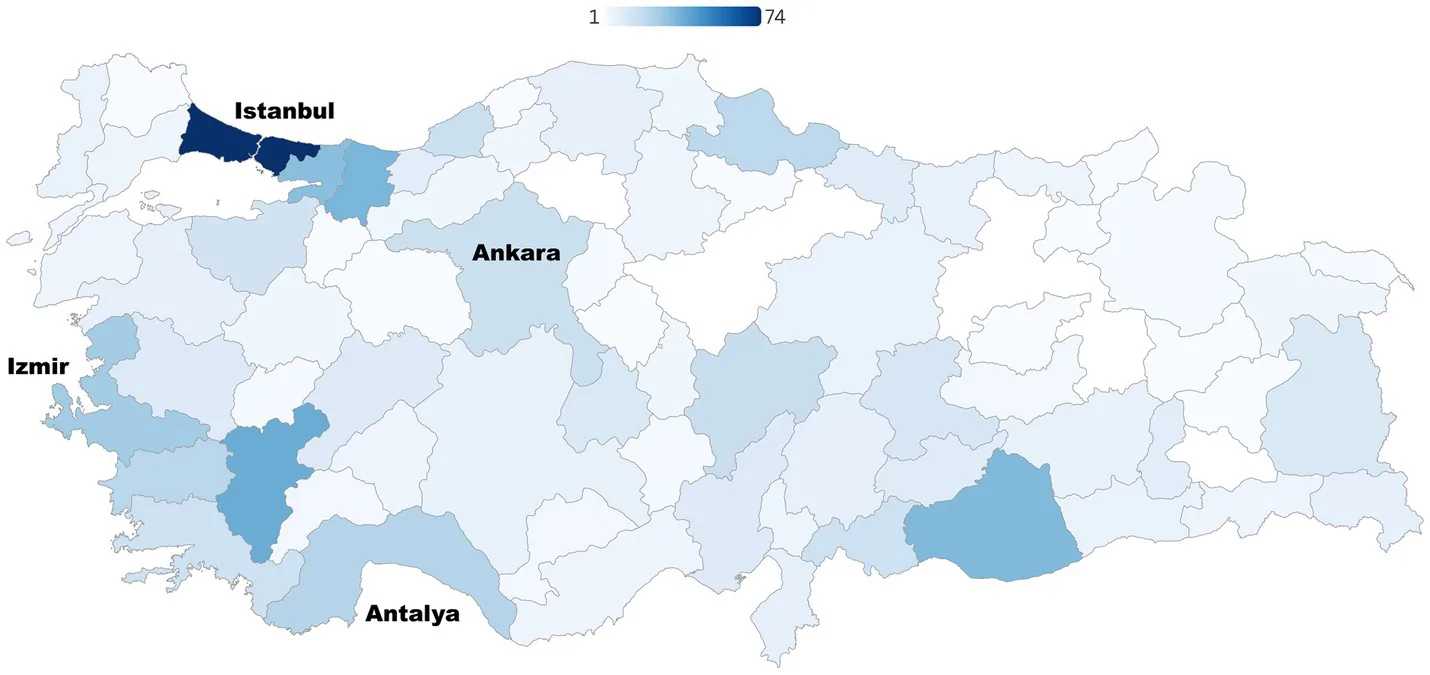
Geographic distribution of media-reported suicide cases across Türkiye.

**Figure 3:**
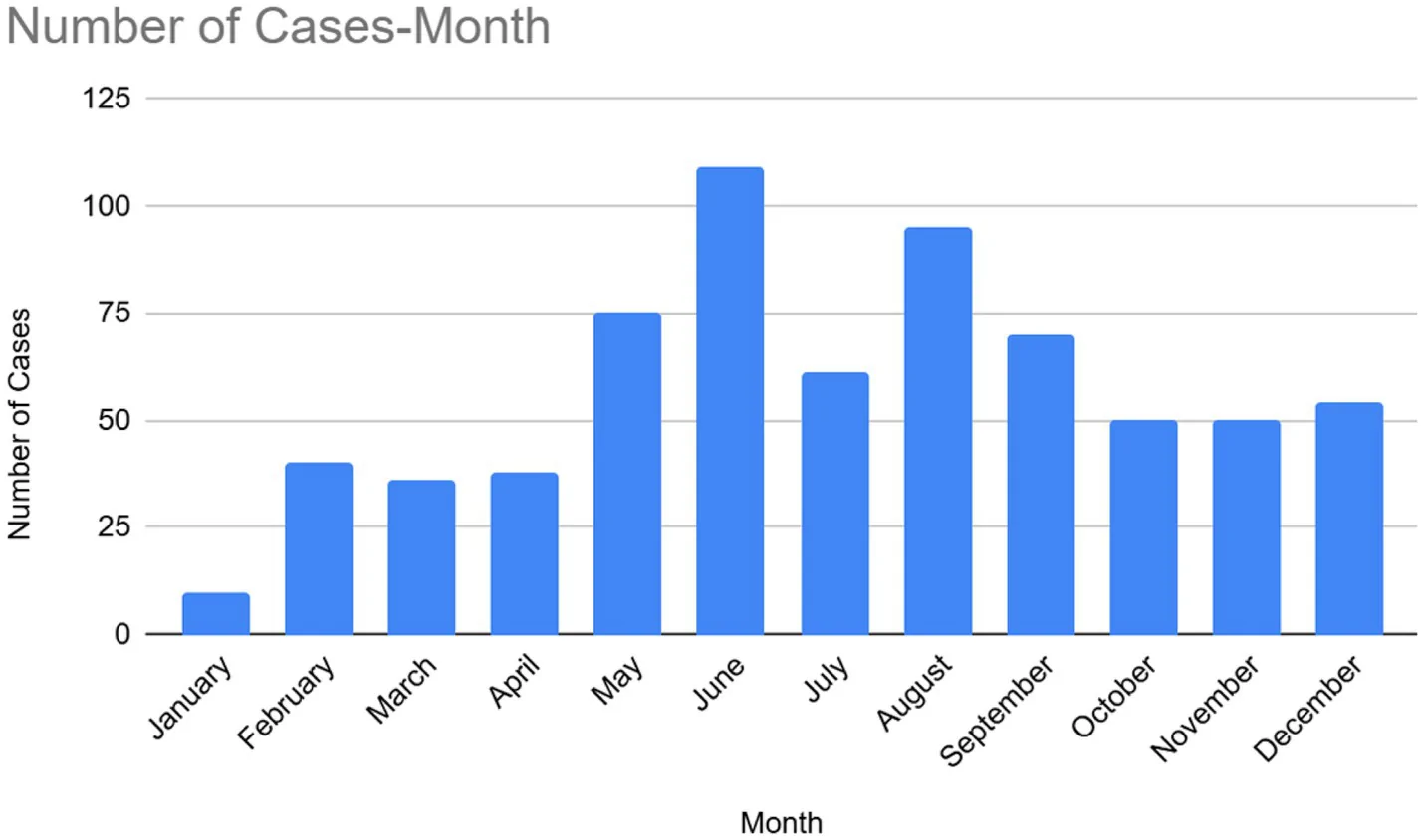
Monthly distribution of media-reported suicide cases during 2025.

**Table 3:** Distribution of suicide methods by sex.

Method	Male *n* (%)	Female *n* (%)	Not reported *n* (%)	Total (*n*)	*p*-value
Firearms	262 (49.6)	19 (17.6)	16 (30.8)	297	<0.001^‡^
Hanging	149 (28.2)	43 (39.8)	17 (32.7)	209	
Jumping from height	22 (4.2)	20 (18.5)	4 (7.7)	46	
Drowning	7 (1.3)	4 (3.7)	0 (0.0)	11	
Sharp instrument	9 (1.7)	3 (2.8)	0 (0.0)	12	
Chemicals	7 (1.3)	2 (1.9)	1 (1.9)	10	
Train/vehicle	8 (1.5)	4 (3.7)	6 (11.5)	18	
Gas (LPG, etc.)	2 (0.4)	0 (0.0)	0 (0.0)	2	
Burning	3 (0.6)	0 (0.0)	0 (0.0)	3	
Not reported	59 (11.2)	13 (12.0)	8 (15.4)	80	
Total	528 (100.0)	108 (100.0)	52 (100.0)	688	

^‡^*p* value was calculated using the Fisher–Freeman–Halton exact test because Pearson’s chi-square assumptions were not satisfied owing to sparse expected cell counts.

**Table 4:** Comparison of age according to suicide method.

Method of suicide	*n*	Age (years), Mean ± SD	Median (IQR)	Age range (years)	*p*-value
Hanging	176	36.2 ± 16.2	31 (21)	14–85	0.087^*^
Chemicals	8	37.9 ± 16.5	34 (26.3)	21–69	
Jumping from height	34	30.8 ± 12.0	28.5 (12.8)	16–73	
Drowning	9	39.2 ± 14.7	42 (27.5)	21–62	
Firearms	211	37.7 ± 14.7	35 (20)	14–84	
Burning	2	46.0 ± 15.6	46 (−)	35–57	
Sharp instrument	10	45.9 ± 15.1	46 (18.8)	18–68	
Gas (LPG etc.)	2	32.5 ± 0.7	32.5 (−)	32–33	
Train/vehicle	8	35.6 ± 13.4	32.5 (22)	22–60	
Not reported	47	33.5 ± 12.7	32 (20)	12–72	
^*^Analysis was restricted to cases with complete age data (*n* = 507). *p* value calculated using the Kruskal–Wallis test.					

Method	*n*	Age (years), mean ± SD	Median (IQR)	Age range (years)	*p*-value
Hanging	176	36.2 ± 16.2	31 (21)	14–85	0.017^*^
Firearms	211	37.7 ± 14.7	35 (20)	14–84	
Jumping from height	34	30.8 ± 12.0	28.5 (12.8)	16–73	
Other methods	39	39.9 ± 14.4	40 (26)	18–69	
Not reported	47	33.5 ± 12.7	32 (20)	12–72	

^*^Analysis was restricted to cases with complete age data (*n* = 507). Because several suicide methods had very small sample sizes, a secondary analysis was performed after combining low-frequency methods into a single “Other methods” category. *p* value calculated using the Kruskal–Wallis test.

**Table 5:** Characteristics of victims in homicide–suicide incidents (*N* = 134 victims).

Victim relationship	*n*	%
Spouse	34	25.4
Girlfriend	11	8.2
Child	18	13.4
Romantic partner	8	6.0
Neighbor	8	6.0
Ex-spouse	7	5.2
Ex-partner	5	3.7
Sibling	3	2.2
Friend	3	2.2
Mother	3	2.2
Father	2	1.5
Brother-in-law	2	1.5
Relative by marriage	2	1.5
Colleague	2	1.5
Son	2	1.5
Employee	2	1.5
Ex-fiancé(e)	2	1.5
Mother-in-law	2	1.5
Other	18	13.4
Total	134	100.0

Variable	Category	*n* (%)
Male	28	20.9
Female	97	72.4
Not reported	9	6.7

Some homicide–suicide incidents involved multiple victims; therefore, the total number of victims (*n* = 134) exceeds the number of homicide–suicide incidents (*n* = 111).

### 3.1 Qualitative findings

Among the 688 suicide cases included in the study, contextual information regarding possible reasons or circumstances surrounding suicide was identified in 179 cases. After all available media reports referring to the same suicide event had been reviewed together, a single case-level contextual narrative was developed for each of these 179 cases. These case-level narratives were inductively coded and organized into five mutually exclusive primary analytic categories comprising one descriptive event/legal category and four contextual themes (Table 1). Each case was assigned to the single analytic category that best reflected its dominant contextual meaning. The descriptive event/legal category, violence, homicide–suicide event characteristics, and legal circumstances, was the most frequently identified analytic category (*n* = 57, 31.8%), followed by physical and psychological health problems and addictions (*n* = 47, 26.3%), family and intimate relationship conflicts (*n* = 44, 24.6%), and economic and occupational difficulties (*n* = 28, 15.6%). Social exclusion and social vulnerability was the least frequently identified theme (*n* = 3, 1.7%).

### 3.2 Descriptive event/legal category: violence, homicide–suicide event characteristics, and legal circumstances

This descriptive event/legal category represents a descriptive category encompassing violence-related event characteristics and legal circumstances reported in the media rather than antecedent causes of suicide. It includes descriptions of homicide–suicide events, domestic violence, criminal investigations, imprisonment, and legal proceedings. Accordingly, the frequencies reported for this category should be interpreted as the occurrence of these reported event characteristics and legal circumstances, not as the frequency of antecedent contextual factors preceding suicide.

Violence-related event characteristics and legal circumstances constituted the largest descriptive category identified in the media reports. Narratives frequently described homicide–suicide events together with domestic violence, criminal investigations, imprisonment, and ongoing legal proceedings. Many reports involved individuals who killed a spouse, intimate partner, family member, or another person before dying by suicide.

Representative quotations included:

“After killing his wife, he ended his own life using the same firearm.”

“The individual arrested on suspicion of murder died by suicide.”

“The individual who was on temporary leave from prison died by suicide.”

#### 3.2.1 Theme 2: Physical and psychological health problems and addictions

The second most frequently identified contextual theme encompassed mental health problems, physical illnesses, and addiction-related difficulties reported in the media. Narratives commonly referred to depression, psychological distress, chronic physical illnesses, substance use, and gambling-related financial difficulties as circumstances surrounding suicide events.

Representative quotations included:

“He was reported to have been experiencing psychological problems.”

“He had reportedly been receiving treatment for depression for a prolonged period.”

“The individual undergoing cancer treatment was reported to have died by suicide.”

“He was reported to have accumulated substantial debt due to online gambling.”

#### 3.2.2 Theme 3: Family and intimate relationship conflicts

Family-related and intimate relationship problems constituted another major theme. Divorce, separation, relationship breakdown, abandonment, and bereavement were frequently described alongside suicide events.

Representative quotations included:

“He was reported to have become emotionally distressed after his girlfriend wanted to end the relationship.”

“The individual whose spouse had left him was reported to have died by suicide.”

“He was reported to have become emotionally distressed after separating from his partner.”

#### 3.2.3 Theme 4: Economic and occupational difficulties

Economic and occupational difficulties constitute another major contextual theme. Media reports frequently describe financial hardship, debt burden, unemployment, job insecurity, and workplace-related conflicts as circumstances surrounding suicide events.

Representative quotations included:

“He was reported to have experienced severe emotional distress due to an inability to repay his debts.”

“He died by suicide after becoming unemployed.”

“He was reported to have experienced psychological distress due to problems at his workplace.”

#### 3.2.4 Theme 5: Social exclusion and social vulnerability

Although less frequently identified, social exclusion and social vulnerability constitute the fifth contextual theme. Media reports described experiences of social exclusion, stigmatization, and distress associated with social media exposure as circumstances surrounding suicide events.

Representative quotations included:

“He was reported to have experienced significant distress following the circulation of images on social media.”

“He was reported to have felt socially excluded by those around him.”

### 3.3 Integration of quantitative and qualitative findings

The qualitative findings complemented the quantitative analyses by providing media-reported contextual information surrounding suicide events. While the quantitative findings demonstrated high rates of firearm use and homicide–suicide, the qualitative analysis identified Violence, Homicide–Suicide Event Characteristics, and Legal Circumstances as the largest descriptive category in media reports. This category included descriptions of homicide–suicide events together with domestic violence, criminal behavior, and legal circumstances reported in the media and should not be interpreted as representing verified antecedent causes of suicide. Psychological distress, family and intimate relationship conflicts, and economic and occupational difficulties also emerged as recurrent contextual themes. Together, these findings indicate that media reports commonly describe suicide alongside a range of psychological, social, economic, and relational circumstances, without establishing their causal or temporal roles.

## 4 Discussion

This mixed-methods study examined suicide cases reported in Turkish media and provided a comprehensive assessment of their epidemiological characteristics, homicide–suicide patterns, and media-reported contextual narratives. Several important findings emerged. First, suicide cases were predominantly male, and firearms represented the most frequently reported suicide method. Second, homicide–suicide accounted for a substantial proportion of cases, and suicide method was significantly associated with homicide–suicide status, with 30.6% of firearm-related suicides involving homicide–suicide. Third, qualitative analysis identified Violence, Homicide–Suicide Event Characteristics, and Legal Circumstances as the largest descriptive category in media reports, while psychological distress, family and intimate relationship conflicts, and economic and occupational difficulties emerged as the most frequently reported contextual themes. Together, these findings reinforce the multifactorial nature of suicide and demonstrate the potential value of media-based surveillance for identifying media-reported contextual information and event characteristics that may not be captured by routine mortality statistics (2–4).

The predominance of male suicide cases observed in this study is consistent with both global and national evidence demonstrating higher suicide mortality among men (8, 11). However, recent national time-trend evidence indicates that sex-specific patterns of suicide mortality in Türkiye are not static. Although crude suicide mortality rates among males increased consistently from 2007 to 2022, rates among females shifted from a declining trend to a significant increase during 2019–2022, with particularly marked recent increases among young adults (12). Therefore, the male predominance observed in the present media-reported sample should not obscure emerging increases among females in official mortality data. International studies have repeatedly shown that men are more likely to die by suicide despite women often reporting higher levels of suicidal ideation and non-fatal suicidal behavior (5). This disparity has been attributed to multiple factors, including greater use of highly lethal suicide methods, lower help-seeking behavior, social expectations regarding masculinity, and increased exposure to occupational and financial stressors (6). Our qualitative findings further identified economic hardship, occupational difficulties, and relationship-related stressors as recurrent themes, consistent with previous evidence that socioeconomic disadvantage and financial strain contribute to suicide vulnerability among men (6, 7).

A particularly important finding was the high prevalence of firearm-related suicides. Suicide method was significantly associated with homicide–suicide status. Firearms accounted for more than two-fifths of all reported suicides, and 30.6% of firearm-related suicides involved homicide–suicide. This finding is consistent with international research demonstrating that access to highly lethal methods substantially increases suicide mortality (2). However, this finding should be interpreted with caution because the present study was based on media-reported suicide cases rather than official mortality records. Compared with official Turkish Statistical Institute (11) mortality statistics, in which hanging is the most common method of suicide and firearm-related suicides account for a smaller proportion of deaths, firearms were overrepresented in the present media-based sample. This discrepancy likely reflects media selection and reporting bias, as violent, unusual, and homicide–suicide events are more likely to receive extensive media coverage than other suicide deaths. Therefore, the epidemiological patterns observed in this study should be interpreted as characteristics of media-reported suicide cases rather than of all suicide deaths occurring in Türkiye. Nevertheless, media-based surveillance provides valuable contextual information that complements official mortality statistics by capturing circumstances that are not routinely available in national surveillance systems. Because this study did not measure firearm availability, ownership, or access, it cannot establish the role of firearm access in the observed events. Nevertheless, the high representation of firearms among media-reported suicide and homicide–suicide cases is consistent with broader evidence linking firearm availability to lethal violence involving intimate partners and family members (23). Accordingly, considerations regarding lethal-means restriction and firearm safety are grounded in the broader evidence base and WHO recommendations rather than presented as causal implications demonstrated by this dataset (2).

One of the most striking findings was the substantial proportion of homicide–suicide cases. Approximately one in six suicide events involved the death of another individual, and homicide–suicide was significantly more common among men than women. In addition, spouses, children, and intimate partners were the most frequently reported victims. These findings are consistent with previous reviews indicating that homicide–suicide incidents most commonly occur within intimate partner and family contexts and are frequently associated with relationship breakdown, domestic violence, and firearm access (23). Consistent with these findings, the qualitative analysis identified Violence, Homicide–Suicide Event Characteristics, and Legal Circumstances as the largest descriptive category in media reports. This category included descriptions of homicide–suicide events together with domestic violence, criminal behavior, and legal circumstances reported in the media. These findings should be interpreted as media-reported event characteristics and contextual descriptions rather than verified antecedent causes of suicide. Together, these findings suggest that homicide–suicide should be viewed not only as a suicide-related phenomenon but also as an important form of interpersonal violence requiring integrated prevention approaches (23).

The qualitative findings revealed that Violence, Homicide–Suicide Event Characteristics, and Legal Circumstances constituted the largest descriptive category identified in the media reports, followed by Physical and Psychological Health Problems and Addictions, Family and Intimate Relationship Conflicts, and Economic and Occupational Difficulties. Because this category included descriptions of homicide–suicide events in addition to violence-related and legal circumstances, its frequency should not be interpreted as representing the frequency of antecedent contextual factors preceding suicide. Complementary community-based evidence from Türkiye has identified a weak positive association between retrospectively reported childhood violence experiences and suicide-probability scores among adults aged 18–40 years (24). However, this evidence concerns self-reported non-fatal suicidality and differs conceptually from the event-level violence, homicide–suicide characteristics, and legal circumstances described in the present media reports. The remaining themes align with contemporary public health models conceptualizing suicide as a multifactorial outcome arising from interactions between psychological vulnerability and broader social determinants of health (3, 4). Relationship-related conflicts, economic hardship, and unemployment were frequently described in media narratives, consistent with previous evidence linking financial strain, debt burden, and socioeconomic disadvantage to suicidality and suicide risk (6, 7).

The present findings also have important implications for media reporting practices. Previous studies have demonstrated that detailed descriptions of suicide methods, sensationalized reporting, and repetitive coverage may contribute to harmful reporting effects and increases in suicidal behavior, commonly referred to as the Werther effect (13). Conversely, responsible reporting practices emphasizing coping strategies and help-seeking behaviors may contribute to protective effects, known as the Papageno effect (16). Recent evaluations of Turkish media have suggested that suicide reporting does not always fully comply with WHO recommendations, highlighting the need for continued monitoring and improvement of reporting practices (14, 15). Given that the present study relied on media reports as a surveillance source and analyzed media-reported contextual information rather than verified causes of suicide, improving the quality and responsibility of suicide reporting may enhance both the value of media-based public health surveillance and suicide prevention efforts.

### 4.1 Strengths and limitations

This study has several strengths. First, it employed a convergent mixed-methods design that integrated quantitative epidemiological analyses with qualitative document analysis, enabling a more comprehensive understanding of suicide than would be possible through either approach alone. Second, the study included a large nationwide sample of 688 media-reported suicide cases collected over a one-year period, allowing the examination of demographic characteristics, suicide methods, regional patterns, and homicide–suicide events. Third, the integration of quantitative and qualitative findings provided important insights into the contextual circumstances and media-reported narratives surrounding suicide, including violence, homicide–suicide event characteristics, psychological distress, relationship conflicts, and economic and occupational difficulties.

Several limitations should also be acknowledged. First, the study relied exclusively on media reports rather than official mortality, forensic, or clinical records. Consequently, reporting bias, selective coverage, and incomplete information may have influenced the findings. Furthermore, because media surveillance relied on predefined Google Alerts search terms and Google’s proprietary indexing algorithm, some relevant media reports may have been missed, and the search strategy may not be fully reproducible. Second, contextual factors described in news reports reflect journalistic interpretations and reported narratives rather than verified causal explanations for suicide. Moreover, contextual information was available for only 179 of the 688 cases, and this qualitative subset was unlikely to be random. Reports containing richer narratives, including homicide–suicide or other unusual events, may have received more extensive coverage; therefore, the qualitative findings may be affected by within-sample selection bias. Third, some demographic and event-related variables were missing in a proportion of cases, limiting the completeness of the analyses. Regional comparisons also lacked population denominators, official region-specific suicide counts, and measures of regional media coverage intensity; thus, the observed regional percentages should not be interpreted as incidence or population-level risk estimates. Fourth, because media outlets are more likely to report unusual, violent, or sensational events, homicide–suicide and firearm-related incidents may be overrepresented compared with official mortality statistics. Consequently, the study sample cannot be considered representative of all suicide deaths occurring in Türkiye. The epidemiological patterns observed in this study should therefore be interpreted as reflecting media-reported suicide cases rather than national suicide epidemiology. Media-based surveillance should be regarded as complementary to official mortality surveillance, providing contextual information that is not routinely captured in official datasets. Finally, only incidents resulting in death were included; cases involving non-fatal self-harm or non-fatal injuries to others were not examined.

### 4.2 Implications for public health practice and policy

The high representation of firearm-related cases in this media-reported sample, considered alongside the broader evidence base and WHO recommendations, supports continued consideration of lethal-means restriction, firearm safety policies, and targeted risk assessment. However, the present data do not directly assess firearm availability, ownership, access, or policy effects. In addition, the frequent reporting of domestic violence, relationship conflicts, and homicide–suicide event characteristics, when interpreted alongside established evidence, is consistent with integrating suicide prevention efforts with violence-prevention and family-protection programs.

Beyond violence-related event characteristics, the recurrent identification of psychological distress, depression, chronic illness, unemployment, debt burden, and social adversity in media reports further highlights the importance of addressing the social determinants of mental health within national suicide prevention strategies. Multisectoral interventions involving healthcare services, social support systems, educational institutions, and community organizations may be necessary to address the complex contextual circumstances surrounding suicide.

The study also highlights the importance of responsible media reporting. Given the potential influence of media coverage on suicidal behavior, greater adherence to WHO guidelines may contribute to safer reporting practices while simultaneously improving the quality of information available for public health surveillance. Training programs for journalists and media professionals may therefore represent an important component of suicide prevention efforts in Türkiye.

## 5 Conclusion

This mixed-methods study provides a comprehensive examination of suicide cases reported in Turkish media and demonstrates the value of integrating epidemiological surveillance with qualitative document analysis. Suicide cases were predominantly male, firearms were the most frequently reported method, and homicide–suicide represented a substantial proportion of reported events. Qualitative analysis identified Violence, Homicide–Suicide Event Characteristics, and Legal Circumstances as the descriptive event/legal category, while psychological distress, family and intimate relationship conflicts, economic and occupational difficulties, and social vulnerability constituted four contextual themes.

The integration of quantitative and qualitative findings indicates that psychological, social, economic, and relational circumstances were reported in association with suicide and homicide–suicide events; these media-described circumstances should not be interpreted as verified causal influences. Although media-based surveillance cannot replace official mortality systems, it can provide valuable media-reported contextual information and event descriptions that complement official data and enhance understanding of suicide. Future research should integrate media reports with official mortality, forensic, and healthcare data to develop a more comprehensive understanding of suicide patterns in Türkiye. Consistent with the broader evidence base and WHO recommendations, strengthening surveillance systems, promoting responsible media reporting, considering measures to reduce access to lethal means, and integrating suicide prevention with domestic violence prevention initiatives may contribute to reducing suicide-related mortality and improving public health outcomes.

## Data Availability

The raw data supporting the conclusions of this article will be made available by the authors, without undue reservation.
